# Development of a duplex qPCR assay with locked nucleic acid probes for A, B and E kappa-casein variants detection

**DOI:** 10.1038/s41598-022-20586-w

**Published:** 2022-09-30

**Authors:** L. Jiménez-Montenegro, J. A. Mendizabal, L. Alfonso, L. Azparren, O. Urrutia

**Affiliations:** IS-FOOD, School of Agricultural Engineering and Biosciences, Public University of Navarre (UPNA), Campus de Arrosadia, 31006 Pamplona, Spain

**Keywords:** Genetic techniques, Sequencing

## Abstract

Milk proteins determine important milk technological characteristics. Among caseins, Ƙ-casein has been correlated with fat and protein content and cheese yield. Fourteen Ƙ-caseins variants have been described but the alleles A, B and E are the most important ones due to their frequency and/or influence on the technological aptitudes of milk. Therefore, in the present study two different duplex qPCR assays with locked nucleic acid probes (for positions 13104 and 13124 of the Ƙ-casein gene) were developed for the detection of A, B and E variants. Firstly, DNA isolation method from milk somatic cells and hair was optimised. The developed 13124-qPCR assay showed an increased sensitivity reaching up to 6.7 copies DNA copies/reaction at a 95% confidence level with A, B and E alleles reference samples. The 13104-qPCR assay reached up to 6.7 DNA copies/reaction for A allele reference sample and 67 DNA copies/reaction for B and E samples. Intra-assay variation results were below 6%. Applicability was determined using DNA samples from animals with known genotype for Ƙ-casein (AA, AB, BB, BE, AE, EE) and both assays were able to discriminate among the six genotypes with 100% accuracy. Thus, this qPCR method represents a sensitive and rapid option for the detection of Ƙ-casein alleles in both hair and milk samples.

## Introduction

Milk proteins play a key role in the determination of the nutritional and technological properties of milk. Processing capabilities of milk during the industrial manufacture of dairy products are determined by milk proteins. This is especially true for caseins, which play a crucial role in rennet formation during cheese production^1^.

Caseins, which account for 80–85% of milk proteins^2^, can be divided into four types: alfa-s1-casein (α-s1-casein), alfa-s2-casein (α-s2-casein), beta-casein (β-casein) and kappa-casein (Ƙ-casein) and they are codified by a group of genes located on the locus *CN* of chromosome 6 (*CSN1S1, CSN1S2, CSN2* and *CSN3,* respectively). Among caseins, Ƙ-casein constitutes a 12% of the total casein contained in bovine milk^3^ and it has been widely studied because its association with qualitative composition of milk^4^, milk yield^5,6^, milk protein characteristics^7^, milk fat yield^8^ and cheese yield^3^. Ƙ-casein stabilizes casein micelles, avoiding its aggregation, and consequently keeping calcium phosphate in solution^7,9–11^. Ƙ-casein protein is encoded by *CSN3* gene^2^. Fourteen Ƙ-caseins variants have been described, but alleles A, B and E could be the most important ones due to their frequency and/or influence on the technological aptitudes of milk. A and B are the most frequent variants of Ƙ-casein in numerous populations of the Friesian breed^12–14^. The estimated allele frequencies for Friesian populations worldwide are 0.520 for the A allele, 0.335 for the B allele and 0.139 for the E allele^13^. Although Ƙ-casein E is less frequent, it has been correlated with detrimental effects on milk technological properties and it is worth to analyse^2,12^.

Ƙ-casein B allele differs from A in two single nucleotide polymorphisms (SNP) on the *CSN3* gene causing amino acids substitution in both 136 and 148 positions. The threonine (Thr) in position 136 and aspartic (Asp) in position 148 of A allele are replaced by an isoleucine (Ile) and an alanine (Ala) in the B allele^15^, respectively. These double amino-acid substitution works as a diallelic system due to the strong linkage disequilibrium created^11^. On the other hand, a SNP distinguish E variant from A variant and leads to another amino acid substitution: in the position 155 of the amino acid sequence, a serine (Ser) in the A allele is replaced by a glycine (Gly) in the E allele^16^.

In numerous studies, Ƙ-casein casein B allele has been correlated with higher protein content in milk, specially casein (weight percentage of casein to total protein)^11,16,17^. It has also been associated with enhanced coagulation properties^11,18^ and higher yield in cheese production^7^. Enhanced coagulation properties refer to a shorter rennet clotting time and higher curd firmness which lead to a reduction in loss of solids during cheese production^11,18^. In contrast, Ƙ-casein A allele has been associated with lower calcium and fat content, as well as, lower pH values in milk^11,18^. Moreover, Ƙ-casein casein E allele, has been suggested to have negative effects on milk coagulation properties and it has been associated with lower concentration of Ƙ-casein^2,12,16^.

Ƙ-casein polymorphisms have received considerable interest among researchers due to the positive effects exerted by the B variant of Ƙ-casein on the cheesemaking process and, hence its potential use in dairy selection programs^7,17,19^. Although there still speculation on incorporating this information into selection programs^20,21^, commercial dairy producers showed little interest because they are not paid for this trait^22^. However, for smallholder dairy farms, which produce raw milk and dairy products on the farm, the analysis of Ƙ-casein could be currently useful to improve the economics value of cheese making^23^.

A wide range of techniques have been previously used for cattle genotyping, such as *Restriction Fragment Length Polymorphism-PCR (RFLP-PCR)*^19^, *Single Stranded Conformation Polymorphism-PCR* (SSCP-PCR)^24^,* Amplification-Refractory Mutation System-PCR* (ARMS-PCR)^11,25^, *Allele Specific-PCR* (AS-PCR)^26^ or *High Resolution Melting Analysis*-qPCR (HRMA-qPCR)^27–29^. Moreover, qPCR assays are highly specific and sensitive DNA-based methods with special interest for genotyping cattle breeds. In other preliminary studies, qPCR protocols were assayed for *CSN3* allelic polymorphisms identification^4,7^, but unlike the present study, a validation of the technique was not developed, in terms of efficiency, linear dynamic range, assay variation and repeatability. Additionally, locked nucleic acid (LNA) probes can be employed to increase the specificity of the qPCR assays^30^. LNA probes present higher affinity to the complementary DNA than other types of probes due to the presence of covalent bridges between some base pairs. As a result, LNA probes improve stability and mismatch discrimination^31^. In this context, the objective of the present study was to develop and optimise a duplex real time qPCR assay with LNA probes for genotyping or identifying of *CSN3* variants (alleles A, B and E) in dairy cattle.

## Results and discussion

### DNA isolation from milk somatic cells and hair

The isolation of high quantity and quality DNA is a preliminary step required for DNA-based methods^32,33^. In cattle, to genotype the animals, DNA is normally isolated from peripheral blood leukocytes, different tissues or hair follicles^33^. Additionally, for other purposes such as milk quality control, DNA can be isolated from milk somatic cells of dairy cattle. However, inhibitors in milk such as fats or proteins render it difficult source for obtaining high yield and purity DNA samples^33,34^. Qualitative and quantitative analyses of milk and hair samples^35,36^ are shown in Table 1.

**Table 1:** Effect of both sample pre-treatment and matrix type on the quantity (ng/μL), quality (A_260/280_) and qPCR results (Cq values) of DNA samples.

Item	Milk (4 °C)	Milk (− 20 °C)	Milk (− 80 °C)	Hair	*P* value
Concentration, ng/µL	13.07 ± 3.01^ab^	9.69 ± 2.88^b^	20.99 ± 0.71^a^	10.74 ± 2.73^b^	0.009
Quality (A_260/280_), nm	1.36 ± 0.06^c^	1.71 ± 0.05^b^	1.92 ± 0.01^a^	1.93 ± 0.05^a^	0.000
Cq value^1^	24.62 ± 0.52^a^	23.30 ± 0.23^a^	18.15 ± 1.40^b^	26.26 ± 0.3^a^	0.000

Cq = Cycle number of crossing quantity (target *CSN2* Table S1).

Values are means ± standard error.

^a,b,c^Means with different lowercase subscript were statistically different to each other (*P* ≤ 0.05; Tukey's test).

^1^Lower values are associated with higher quantities of amplifiable DNA.

Regarding DNA concentration, results indicated that isolating DNA from milk samples of − 80 °C pre-treatment yielded a significant higher concentration than the other samples types (*P* ≤ 0.05), but not statistically significant with respect to milk refrigerated at 4 °C (*P* > 0.05). The A_260/280_ ratio is used to determine DNA quality. Ratio values between 1.8 and 2 are considered optimal whilst lower values indicated a contamination of aromatic compounds such as phenolics and proteins^33,37,38^. In this sense, results of DNA isolated from milk at − 80 °C and from hair follicles fitted best to A_260/280_ ratio optimal values.

Regarding Cq values, lower values are preferred, because they are associated with higher quantities of amplifiable DNA^35^. In this case, Cq values were adequate in all sample types and no significant differences were observed among them (*P* > 0.05), with the exception of DNA isolated from milk at − 80 °C (*P* ≤ 0.05) that showed a briefly lower Cq mean (18.15) as a consequence of its higher DNA concentration.

Results showed that the pre-treatment of milk samples had an effect on the quality and quantity of the isolated DNA. A lower temperature during the pre-treatment leaded to greater release of DNA from milk somatic cells. This phenomenon, has been observed in other preliminary studies^36^, may be due to a higher mechanical damage caused in tissues and cell walls of somatic cells during the freezing and subsequent thawing process. As a conclusion, DNA isolated from milk of − 80 °C pre-treatment and from hair follicles showed the optimal results. Although similar results were obtained with both matrices, isolation of DNA from hair follicles is the desirable option for animal genotyping rather than from milk somatic cells, because hair samples can be obtained from animals of any gender and age, and there is no needed to be in lactation. In contrast, although the high variability in somatic cells content normally observed^35,36^, bulk milk samples should be considered if cheese manufactures or dairy industry in general, required certain Ƙ-casein composition information.

### qPCR validation for detection of A1 and A2 alleles of the β-casein

#### Amplification efficiency and linear dynamic range

**Figure 1:**
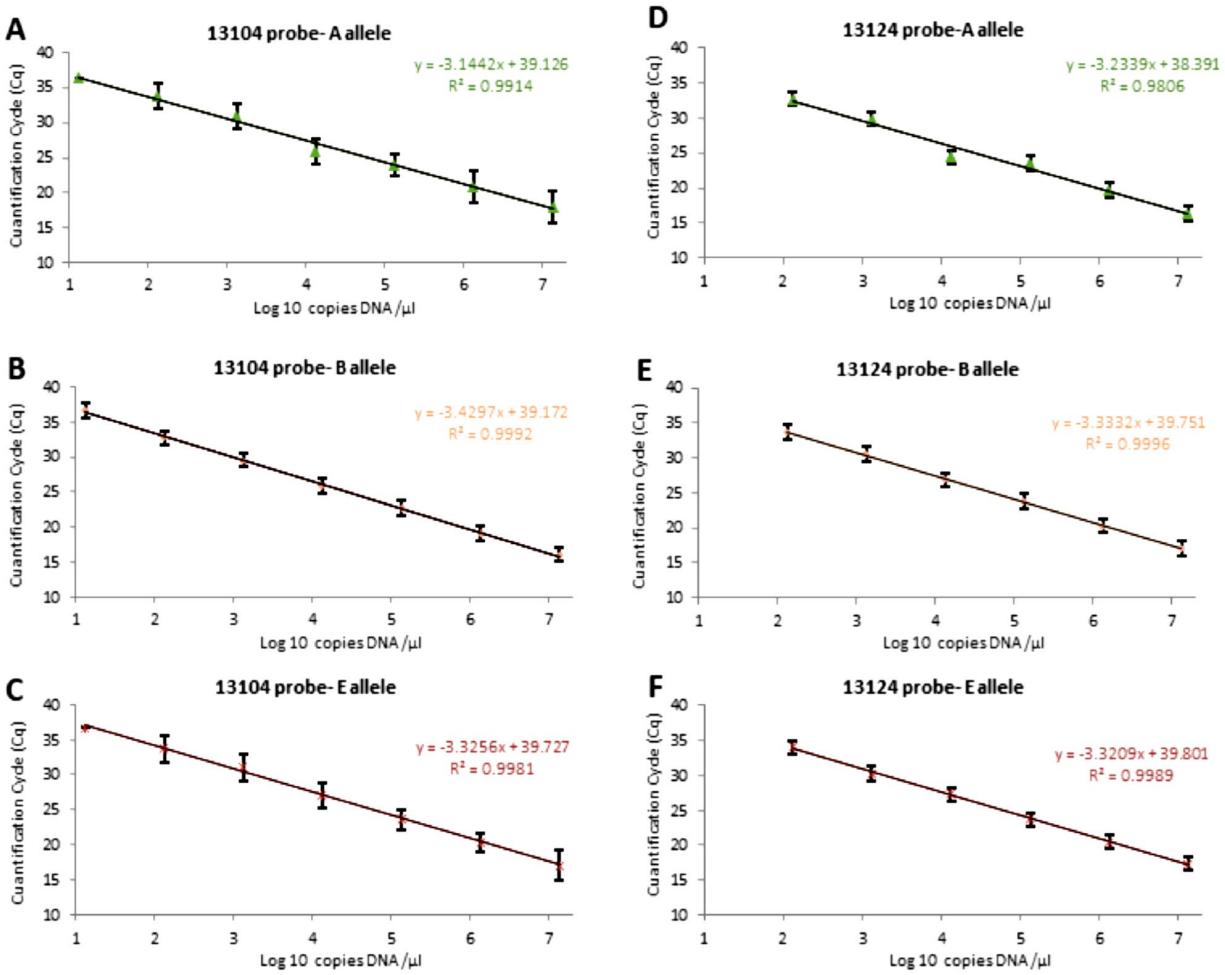
Duplex qPCR standard curves using synthetic reference DNA samples. On the left side, 13104-qPCR assay using A reference sample (**A**), B reference sample (**B**) and E reference sample (**C**). On the right side, 13124-qPCR assay using A reference sample (**D**), B reference sample (**E**) and E reference sample (**F**). Average values, and standard deviations of the three runs carried out are shown. Linear equations and coefficient of determinations (R^2^) are detailed.

The six standard curves required for the validation of duplex qPCR assay for A, B and E allele discrimination of the *CSN3* gene are presented in Fig. 1. Efficiency values are one of the most important indicators of the performance of a qPCR assay and are required to develop a validation protocol. Efficiency in the duplex 13104-probed based qPCR revealed an averaged value of 102.5% for A allele (R^2^ = 0.991), 96.91% for B allele (R^2^ = 0.999) and 97.17% for E allele (R^2^ = 0.998), both through FAM reporter detection (Fig. 1A,B,C).

Similar to other preliminary studies^39^, a standard curve with an AA homozygous genotyped DNA sample was employed to verify the correlation with respect to a synthetic DNA sample (the A synthetic reference DNA sample) (Supplementary Fig. S1). Efficiency of the 13104-probed based qPCR using the homozygous AA genotyped DNA sample was 106.61% (R^2^ = 0.940) through FAM reporter detection, consequently showing an accepted efficiency value^40^ and similar results with respect to obtained with the A synthetic reference sample (102.5% and R^2^ = 0.989).

Efficiency in the duplex 13124-probed based qPCR was in average 103.9% (R^2^ = 0.981) for A allele and 99.54% for B allele (R^2^ = 0.999) through Cy5 reporter detection and E allele average efficiency resulted in a 100.10% (R^2^ = 0.999) through FAM reporter detection (Fig. 1D,E,F). Thus, the efficiency showed adequate values, which proves a good performance of the primer–probe sets^41^. Moreover, the linearity (R^2^) of the standard curves ranging from 0.939 to 0.999 meet the accepted criterion of R^2^ at around 0.98^42^ and, consequently, showed a linear correspondence among log_10_ gene copy number and their respective Cq values.

Apart from that, linear dynamic range of both 13104 and 1324-probed based qPCR assays with A, B and E synthetic reference DNA samples were in all cases from 6.7 × 10^6^ to 67 DNA copies/reaction. Moreover, linear dynamic range with the AA genotyped DNA sample ranged from 4440 to 69.4 DNA copies/reaction.

#### Limit of detection (LOD) and limit of quantification (LOQ)

The 13104-probed based qPCR assay reached up to a LOD of 6.7 DNA copies/reaction for A allele and a LOD of 67 DNA copies/reaction for both B and E alleles. In other validation qPCR protocols, not involving *CSN3* gene, a lower gene copy number was detected, because the degree of dilution made was higher and consequently the starting DNA copy number was lower than that developed in this study. For example, Baudy et al.^41^ reached three DNA plasmid copies/reaction or Forootan et al.^43^ reached 2.7 DNA copies/reaction. On the other hand, the 13124 probe-based qPCR assay revealed more accuracy and a LOD of 6.7 DNA copies per reaction with standard curve A, B, and E was obtained. Respect to LOQ, an equal value as the minimum target DNA of the linear dynamic range was calculated in both duplex qPCR assays (67 DNA copies/reaction)^44^. Results showed an adequate LOD and LOQ values and a low amount of DNA was detectable and quantifiable.

#### Precision: intra-assay and inter-assay repeatability

The 13104-probe qPCR intra-assay variability showed a Coefficient of Variation % (CV) of Cq values that ranged from 0.97 to 2.83% for the synthetic A DNA sample, from 0.51 to 0.72% in the case of synthetic B DNA sample and from 0.71 to 1.69% for synthetic E DNA sample. In the case of the inter-assay variability the mean CV was 3.22% for A allele, 0.84% for B allele and 2.97% for E allele. On the other hand, the 13124-probe qPCR intra-assay repeatability revealed an averaged CV of Cq values that ranged from 0.35 to 2.09% for the synthetic A DNA sample, from 0.29 to 0.42% for the synthetic B DNA sample and from 0.89 to 1.50% for the synthetic E DNA sample. The inter-assay repeatability of this duplex assay showed an averaged CV value of 1.55% for the A allele, 1.16% for the B allele and 1.74% for the E allele. The detailed results are shown in Supplementary Material (Table S1).

For a correct validation of a qPCR protocol, CV values less than 25% are needed for both intra and inter-assay repeatability^45^. In this sense, the present study met the requirements established by the FDA and showed consistent standard curves, which is particularly important to accurately detected A, B and E alleles for cattle genotyping.

### Applicability of the duplex qPCR assay to Ƙ-casein alleles identification

**Figure 2:**
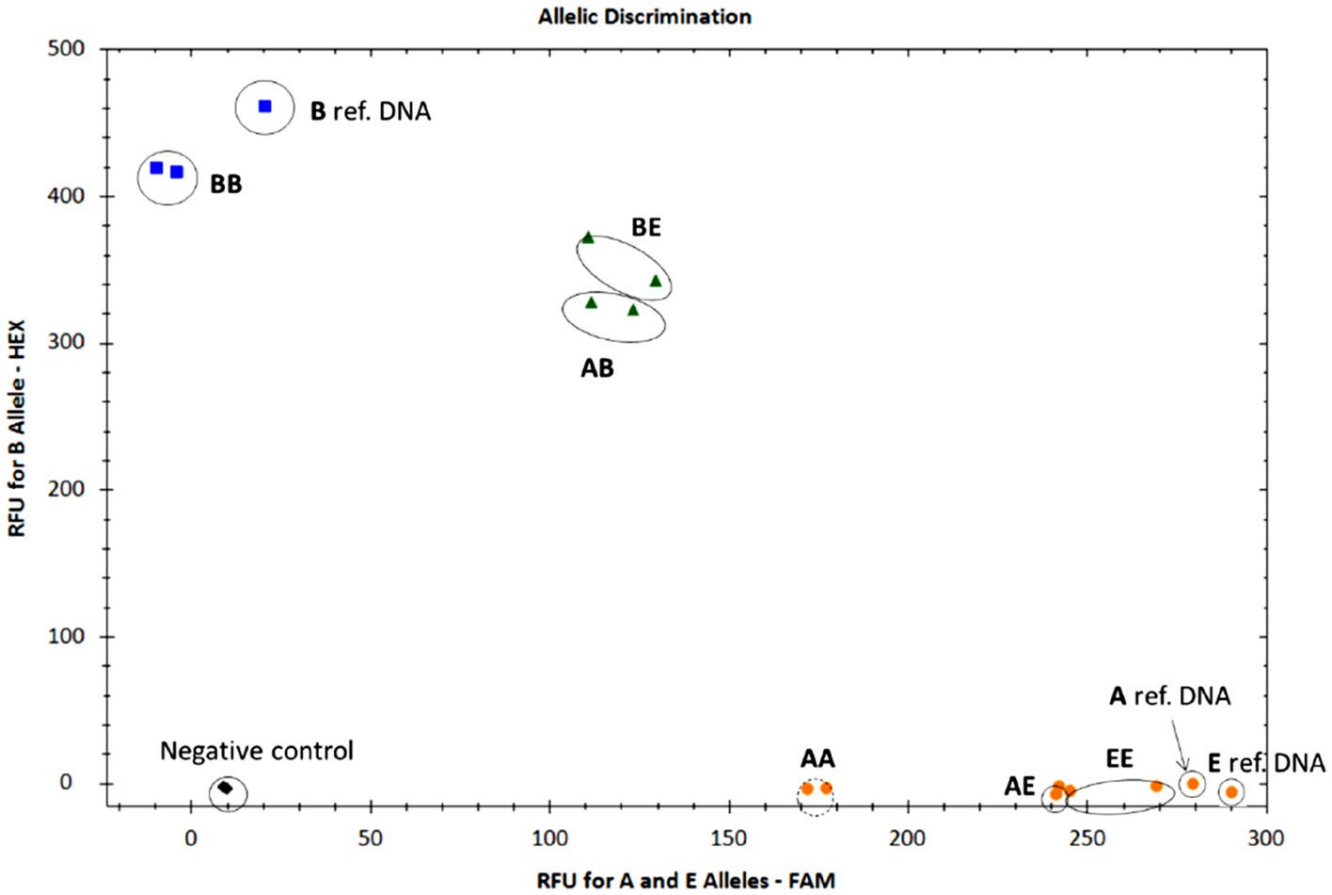
Allelic discrimination graph of 13104-probe qPCR assay for AA, AB, BB, BE and EE genotyped DNA samples from hair follicles of genotyped animals. Nonspecific amplifications were not observed. The Blue dots represent BB genotype (HEX reporter), the greens dots represent heterozygous AB or BE genotypes, the yellow dots represent the heterozygous AE and the homozygous AA and EE genotypes (FAM reporter); the black dots represent negative control (NTC). A, B and E synthetic reference samples were used as a positive control of the assay. Data were plotted using RFU of the FAM reporter dye of A and E alleles on the x axis and using RFU of the HEX reporter dye of B allele on the y axis. RFU = relative fluorescence units.

**Figure 3:**
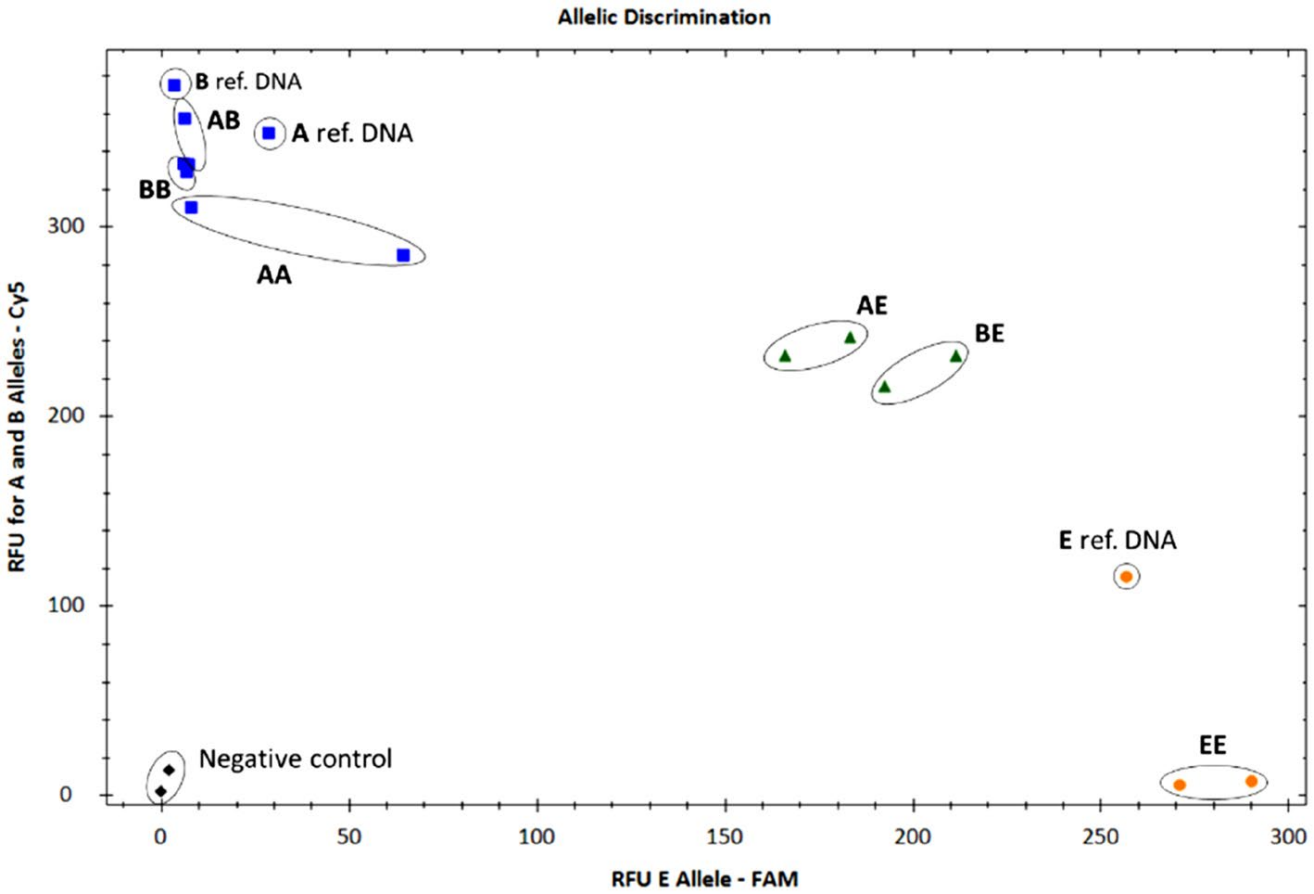
Allelic discrimination graph of 13124-qPCR assay for AA, AB, BB, BE and EE genotyped DNA samples from hair follicles of genotyped animals. The blue dots represent the heterozygous AB genotype and the homozygous AA and BB genotypes (Cy5 reporter), the greens dots represent heterozygous AE or BE genotypes, the yellow dots represent the homozygous EE genotype; the black dots represent negative control (NTC). A, B and E synthetic reference samples were used as a positive control of the assay. Data were plotted using RFU of the FAM reporter dye of E allele on the x axis and using RFU of the cy5 reporter dye of A and B allele on the y axis. RFU = relative fluorescence units.

Results of applicability with each duplex qPCR assay using DNA from hair follicles of genotyped animals are shown in Figs. 2 and 3. The same analysis using DNA from milk of − 80 °C pre-treatment of genotyped animals is shown in Supplementary Fig. S2 and S3.

The duplex qPCR assays for 13104 and 13124 positions worked correctly and all the genotypes were detected (AA, AB, BB, BE, EE) using both DNA from hair follicles and from milk samples. There was 100% agreement with the preliminary genotyping results of the analysed animals. For Ƙ-casein allele detection, a qPCR assay using fluorescent-labelled primers was also developed by Vafin and Gilmanov^7^, in which amplification curves were used to discriminate among AA, AB and BB genotypes, but E genotypes were not included in the analysis. In this sense, the present study is able to detect more genotypes as it uses two duplex assays with four different probes (13104_A, 13104_C; 13124_A, 13124_G) and three different reporters (FAM, HEX, and Cy5) for A, B and E Ƙ-casein polymorphisms detection.

At this point, few qPCR protocols exist to detect *CSN3* polymorphims and those that have been developed^4,7^ have only been tested to detect A and B alleles but no E allele. In addition, other DNA-based methods have been developed for assessing *CSN3* allelic polymorphisms, but also in all of them either or no protocols have been developed for the detection of the E allele or protocols have only been developed for the detection of one of the SNPs of the B allele^11,19,27^. Representative curves of the allele discrimination are shown in Figs. S4 and S5. Furthermore, qPCR methods are a faster, more sensitive and cost-effective option than the other DNA-based methods previously used^46^. The present study represents the first attempt for the detection of AA, AB, BB, BE, AE, EE genotypes of the *CSN3* gene for cattle genotyping using qPCR assays. However, for the future use of the developed method in a scale-up commercial version a better evaluation of the PCR reaction inhibitors or impurities should be performed as this protocol was evaluated using a limited number of samples. For instance, the measurement of the ratio absorbance at A260 and 230 nm or the use of an internal control in the PCR reaction would be useful.

## Conclusions

The isolation of a high-quality DNA is an essential step for the development of a qPCR assay. In the present study, DNA was isolated from hair follicles as well as from milk samples stored at three different temperatures (4 °C, − 20 °C, − 80 °C), to determine whether the type of matrix and pre-treatment have an effect either on quality or quantity of DNA. Results showed that isolated DNA from hair follicles and from milk of − 80 °C pre-treatment were the most suitable options. Two different duplex qPCR assays for A, B and E Ƙ-casein polymorphisms were optimised and validated for 13104 and 10124 positions of *CSN3* gene. The 13124-qPCR assay showed an increased sensitivity reaching up to 6.7 copies DNA copies/reaction at a 95% confidence level with A, B and E synthetic reference samples. The 13104-qPCR assay reached up to 6.7 DNA copies/reaction for A synthetic reference sample and 67 DNA copies/reaction for B and E synthetic reference samples, at a 95% confidence level. Efficiency values were in the accepted range of 90–110% and repeatability results were below 6%, indicating the consistency and robustness of the standard curves developed. Moreover, applicability analysis indicated that both duplex qPCR assays were enabled to discriminate the six genotypes (AA, AB, BB, BE, AE, EE) with 100% accuracy. In this sense, this approach represents a rapid, a sensitive and a cost-effective option for Ƙ-casein alleles detection and it is conceived as an alternative to other DNA-based methods for cattle genotyping. Furthermore, it could be employed if cheese manufacture required Ƙ-casein genotype information of bulk milk samples.

## Materials and methods

### Sample selection

Different milk, hair and DNA samples were used for this study. Firstly, for the DNA isolation and posterior analyses of DNA samples obtained, 14 samples of fresh commercial skimmed milk were used for each milk pre-treatment considered (− 80 °C, − 20 °C and 4 °C). Moreover 12 hair samples from previously genotyped animals with known genotype for Ƙ-casein gene were used (EuroG MD Microarray).

For the optimisation of qPCR assay (primers and probes), it was used a pool of the 14 DNA samples obtained from milk pre-treated at − 80 °C.

Apart from that, for the validation of duplex qPCR assay, synthetic reference DNA samples which contains the sequence of the *CSN3* gene with the specific allele either A allele, B allele or E allele were used. A sample of DNA extracted from the hair follicle of a homozygous AA animal previously genotyped for Ƙ-casein was also used.

Finally, for applicability of the duplex qPCR assays 12 hair samples and 12 milk samples from previously genotyped animals with known genotype for Ƙ-casein gene were used (EuroG MD Microarray). Two cows from each genotype studied (AA, AB, BB, AE, BE, EE) were considered.

No animals were used, bred or provided in the experiments, only samples provided by a dairy company were used; therefore, the experiment was performed in accordance with regulations of the Ethics, Animal Experimentation and Biosafety Committee of the Public University of Navarra (2007-11-12), and the Spanish national regulation (RD 53/2013), which establishes the basic applicable standards for the protection of animals used in experimentation and other scientific purposes, including teaching^47^.

### DNA isolation

Isolation and purification of DNA are crucial steps in DNA molecular techniques. To this end, two different matrices (milk and hair) were used. Hair samples from two cows of each one of the six genotypes considered (AA, AB, BB, AE, BE, EE) were employed. In addition, milk samples were conserved at three different temperatures (− 80 °C, − 20 °C and 4 °C) no more than 10 days, to elucidate whether the DNA quantity and quality obtained were affected by the pre-treatment^48^.

The number of milk samples required to discern whether the milk pre-treatment was significant in DNA isolation was calculated based on the ratio of absorbance at 260 nm and 280 nm (A_260/280_) using a Nanodrop 2000 spectrophotometer (Thermo Scientific, Madrid, Spain). For the analysis, *pwr.t.test* tool of R statistical package *pwr*^49^ was used in which a two-tailed t-student test for comparison of means was performed with a significance value of 95%, a power of 70%, a 0.2 value for standard deviation (SD) of the A_260/280_ ratio and a difference to be detected of 0.2, according to the reviewed literature^33,36,50^. Based on these requirements, 14 milk samples were considered for the analysis.

DNA samples were isolated according a commercially available kit, *Nucleospin Tissue* (Macherey–Nagel, Düren, Germany). For milk samples, a pre-treatment was required prior to DNA isolation^36^. DNA isolation method for both milk and hair samples is detailed in Supplementary information (S1).

### Analysis of DNA samples isolated from milk somatic cells and hair

After DNA isolation, total DNA quantity and quality of hair and milk samples and the optimal pre-treatment of milk samples (either − 80 °C, − 20 °C or 4 °C) were determined. The DNA quantity (expressed as ng per µL) and purity index (A_260/280_ ratio) were calculated using NanoDrop 2000 spectrophotometer (Thermo Scientific, Madrid, Spain)^35^.

The amplificability of the isolated DNA from both milk and hair samples was evaluated employing a previously optimised protocol for *CSN2* gene, which encodes β-casein protein^51^. An A2 DNA gBlocks Gene Fragments (IDT, Coralville, USA) containing the DNA region of the *CSN2* gene with A2 allele (3.67 × 10^6^ DNA copies) was used as positive control, and nuclease-free water was used as negative control of the reaction^35^. The sequences of both primers and the A2 gBlock are shown in Supplementary information (Table S2). Amplification reaction was carried out using a SYBR green-based qPCR assay in a CFX96 Touch qPCR Detection System and with Multiplate^™^ 96-well PCR plates (BioRad, Munich, Germany). A total reaction volume of 10 µL was required, comprising 5 μL of TB Green Premix Ex Taq II (Tli RNase H Plus) (Takara Bio, Otsu, Japan), 0.3 μM of each primer of the *CSN2* gene (IDT, Coralville, USA), 2 μL of template DNA and 2.4 μL nuclease-free water (Cytiva, Amersham Place, United Kingdom). Thermal cycling conditions were as follows: initial denaturation step at 95 °C for 30 s, followed by 40 cycles including denaturation at 95 °C for 15 s, annealing/extension at 60 °C for 1 min, and finally, amplicon melting curve from 65 to 95 °C (with increments of 0.5 °C/5 s). Dissociation curves were examined for the presence of a single product.

The variables quantity (ng/µL), quality (A_260/280_) and amplificability (Cq) data were analysed by simple one-way analysis of variance (ANOVA) using SPSS 21.0 Statistics (SPSS Inc., Chicago, USA), and multiple comparisons between the four groups were performed with Tukey's test.

### Design of primers and locked nucleic acid (LNA) probes for the duplex qPCR assay

The primers and probes were designed using the "PrimerQuest Tool", available on the website of the commercial company Integrated DNA Technologies (IDT), Inc. (https://sg.idtdna.com/PrimerQuest/Home/) which subsequently synthesised them. For its design, a bibliographic search was initially carried out for the sequence of *CSN3* gene for the *Bos Taurus* species with the DNA sequence database "GenBank" (*Bos Taurus* Ƙ-casein GenBank: AY380228.1) containing the polymorphisms of *CSN3*-A allele (GenBank: AY380228.1), *CSN3* B-allele (GenBank: AY380229.1) and *CSN3*-E allele (Genbank: AF041482.1). During this search, the positions of the mutations in the sequence of the three alleles were identified. Allele B had two mutations with respect to allele A, while allele E had only one. Thus, it was possible to identify a fragment of the gene in which, with relative proximity, one of the polymorphisms of the B allele (position 13104) and the mutation of the E allele (13124) were contained (Table 2). A duplex qPCR assay, for each pair of probes in the same position was developed. The 13014 probes discriminated B-allele (13104_C probe) with respect to A-allele and E-allele (13014_G probe). A HEX reporter dye was used with 13104_C and a FAM reporter dye was employed to label 13104_A probe. Conversely, the 13124 probes discerned E-allele (13124_G probe) with respect to A and B allele (13124_A probe). In this case, a FAM reporter dye was used to label 13124_G probe, and a Cy5 reporter dye was used with 13124_A probe. A schematic representation of the expected results of duplex qPCR assays with each reporter dye in two positions is shown in Table 3.

**Table 2:** Primers and locked nucleic acid (LNA) probes used in the duplex qPCR assay.

Target	Oligonucleotide	Sequence (5′–3′)^1^	Amplicon size (bp)
*CSN3*	Forward primer	AAGTACACCTACCACCGAAG	102
	Reverse primer	GTAACTTGGACTGTGTTGATCTC	
	13104_A (A and E allele)	FAM/AGAA+G+**A**+TT+CT+C+CA	
	13104_C (B allele)	HEX/CTA+CT+CTA+GAA+G+**C**+TT	
	13124_A (A and B allele)	Cy5/TT+A+TTGA+G+**A**+GC+CC′	
	13124_G (E allele)	FAM/ TAT+T+GA+G+**G**+GC	

^1^LNA bases are represented by “+” and the detected polymorphism in bold.

**Table 3:** Schematic representation of the expected results of duplex qPCR assays with each reporter dye in the 13104 and 13124 positions.

Position	Reporter	Ƙ-Casein genotype					
		AA	AB	AE	BB	BE	EE
13104^1^	FAM	**✔**	**✔**	**✔**		**✔**	**✔**
	HEX		**✔**		**✔**	**✔**	
13124^2^	Cy5	**✔**	**✔**	**✔**	**✔**	**✔**	
	FAM			**✔**		**✔**	**✔**

^1^The 13104_C probe for B detection was labelled with HEX reporter dye and the 13104_A probe for A and E detection was labelled with FAM reporter dye.

^2^The 13124_G probe for E detection was labelled with FAM reporter dye and the 13124_A probe for A and B detection was labelled with Cy5 reporter dye.

To verify the correct functioning and design of the primers and LNA probes, an *in-silico* analysis was performed using the IDT OligoAnalyzer tool (https://eu.idtdna.com/pages/tools/oligoanalyzer).

To validate the duplex qPCR assay three synthetic reference DNA gBlocks Gene Fragments (IDT, Coralville, USA) were produced (A_gBlock, B_gBlock and E_gBlock) each containing the amplicon of the *CSN3* gene selected in this study (102 pb), with the specific sequence of either allele (A, B and E) (Supplementary Table S3).

### Optimisation of qPCR assay for A, B and E Ƙ-caseins identification

For this analysis, only DNA from skimmed milk samples were used because of the limited number of hair samples that were available from previously genotyped animals. Firstly, optimal primer concentration for the *CSN3* gene amplification was determined according to other preliminary studies^4,7^. A SYBR green-based qPCR assay was developed. A total reaction volume of 10 μL was used containing 5 μL of TB Green Premix Ex Taq II (Tli RNase H Plus) (Takara Bio, Otsu, Japan), three different primer concentrations (0.2, 0.3, and 0.4 µM) (IDT, Coralville, USA) and different DNA quantities (30 ng in 2 µL, 45 ng in 3 µL and 60 ng in 4 µL). Thermal cycling conditions for *CSN3* gene amplification were: initial denaturation step at 95 °C for 30 s, followed by 40 cycles including a denaturation step at 95 °C for 5 s and an annealing/extension at 60 °C for 30 s. The final step was an amplicon melting curve (65 °C for 5 s and 95 °C during 50 s).

After primer optimisation with SYBR green-based qPCR assay, results were not conclusive enough to select either 0.2 or 0.4 µM. As a result, the optimal primer concentration was assayed again during LNA probes optimisation via singleplex and duplex LNA probe-based qPCR assay, with different thermal cycling conditions. Singleplex qPCR assay was developed with each probe separately (13104_A, 13104_C, 13124_A and 13124_G). Duplex qPCR assays were developed with each pair of probes corresponding at the same gene position: one duplex reaction was developed using 13104-probes and the other one with 13124-probes. To determine optimal LNA probe concentration a total reaction volume of 10 µL was used containing 5 µL of Premix Ex Taq (Probe real-time PCR) 2X (Takara Bio, Otsu, Japan), 2 µL template DNA, different primers and probes concentrations (0.2 µM primer was evaluated using either 0.07 µM or 0.1 µM of each probe, and 0.4 µM primer was evaluated employing 0.2 µM of each probe). In addition, optimal thermal cycling conditions for optimal LNA probes performing was determined through varying annealing step^48,52,53^: different temperatures (58 °C, 59 °C or 60 °C) and different timing (30 s or 45 s) were assessed. Amplification conditions were as follows: initial denaturation step at 95 °C for 30 s, followed by 40 cycles including a denaturation step at 95 °C for 5 s and the different annealing steps previously detailed.

The optimised duplex qPCR assay (10 μL) for both 13104 and 13124-probes, contained 5 µL of Premix Ex Taq (Probe qPCR) 2X (Takara Bio, Otsu, Japan), 0.4 µM forward and reverse primers, 0.2 µM each probe, 2.4 μL nuclease-free water (Cytiva, Amersham Place, United Kingdom) and 2 μL target DNA. All qPCR reactions were performed in a CFX96 Touch qPCR Detection System (BioRad, Munich, Germany) using CFX Maestro™ software. Thermal cycling conditions were as follows: initial denaturation step at 95 °C for 30 s, followed by 40 cycles including a denaturation step at 95 °C for 5 s and the annealing step was fixed in 58 °C (melting temperature, Tm) 30 s for 13104 probes and in 60 °C (Tm) 30 s, for 13124 probes.

### Validation of duplex qPCR assay for A, B and E Ƙ-casein identification

To validate qPCR assays, the amplification efficiency, linear dynamic range, LOD, LOQ and repeatability were evaluated according to the United States Food and Drug Administration (U.S. FDA)^45^ and the Codex Alimentarius^54^ and Burd^55^ guidelines. Synthetic reference DNA samples (gBlocks) of each allele (A, B and E) were used in different assays during qPCR validation protocol. Synthetic reference DNA samples containing A, B and E allele of the *CSN3* gene, respectively, were serially tenfold diluted in nuclease-free water (Cytiva, Amersham Place, United Kingdom) from 6.73 × 10^6^ DNA copies to 6.73 DNA copies per reaction. Additionally, an AA homozygous DNA sample was serially twofold diluted in nuclease-free water (Cytiva, Amersham Place, United Kingdom) from 4440 DNA copies to 69.38 DNA copies per reaction. The details about DNA copy number calculation are shown in Supplementary Material (S2).

Three standard curves were developed with the duplex 13104-probed based qPCR assay and the respective synthetic reference DNA sample (A_gBlock, B_gBlock and E_gBlock) and other three standard curves were constructed with the duplex 13124-probed based qPCR, and also, the respective synthetic reference DNA sample. Therefore, a total of six standard curves were required for a correct qPCR validation. Serial dilutions were analysed in three replicates in three qPCR runs resulting in 9 replicates measurements for each dilution point^45,54^. Additionally, a standard curve with a serially diluted genotyped AA DNA sample was carried out to ensure exhibition of similar properties of probes and primers under optimised conditions. Standard curves were created by plotting the Cq against the log_10_ of the DNA copy number.

#### Amplification efficiency and linear dynamic range

The amplification efficiency refers to the average number of DNA copies per amplification cycle, which can assume values in the range of 90–110%^45^. Efficiency of the duplex 13104-probed based qPCR and duplex 13124-probed based qPCR were determined using the standard curves previously detailed^56^. Thereafter, averaged efficiency and coefficients of determination (R^2^) values of the three runs were obtained.

Additionally, linear dynamic ranges of the qPCR assays were calculated through fitting efficiency to 90–110% and R^2^ parameters at around 0.98^56^ for a proper validation assay^40,56^ and a correct linear dynamic range determination^45,57^.

#### Limit of detection (LOD) and limit of quantification (LOQ)

To determine the LOD and LOQ of each duplex qPCR assay with each respective synthetic reference DNA samples (A, B and E) were assessed. 9 replicates for each sample were used^54^. LOQ parameter is defined as the minimum target DNA quantity that can be trustworthy quantified^44,45^. To establish the LOQ of an assay, the CV of the DNA replicates Cq values must be lower than 0.5 Cq^45^. To this end, CV were calculated according to the formula: CV = standard deviation Cq values/average Cq. Furthermore, the LOQ should be the minimum target DNA concentration included in the linear dynamic range^44^. Moreover, LOD is understood as the minimum target DNA quantity at which a positive result is obtained in the qPCR assay with a probability of at least 0.95 (at a 95% confidence level)^43,45^.

#### Precision: intra-assay and inter-assay repeatability

To determine precision of each duplex qPCR assay, intra-assay and inter-assay repeatability were calculated. Repeatability refers to the precision and robustness of an assay with the same sample replicates analysed under the same conditions (same apparatus, same operator and same laboratory)^48^. Intra-assay variability was assessed considering three replicates in a single run and inter-assay repeatability was determined using three runs performed on different days, with three replicates each. CVs among DNA replicates Cq values were calculated according to the formula previously detailed^56^.

### Applicability of the duplex qPCR assay to Ƙ-casein alleles identification for cattle genotyping

The main applicability of this study is based on the assessment of *CSN3* polymorphisms for cattle genotyping. In particular, identification of the A, B and E Ƙ-casein variants in dairy cattle breeds is sought. For this purpose, the DNA from 12 hair samples and 12 milk samples (pre-treatment − 80 °C) of individual cows with known genotype for Ƙ-casein (AA, AB, BB, BA, AE, EE) were used. Allelic discrimination plots with each duplex qPCR assays and AA, AB, BB, BA, AE, EE genotyped DNA samples were developed. The objective was to determine whether the probes were specific enough to detect A, B and E alleles depending on the DNA genotyped sample used. Each duplex qPCR assay was optimised under specific DNA, primers and probes quantities and thermal cycling conditions. For the duplex 13104-probe qPCR assay, a total reaction volume of 10 µL was used containing 5 µL of Premix Ex Taq (Probe qPCR) 2X (Takara Bio, Otsu, Japan), 0.4 µM of each primer (IDT, Coralville, USA), 0.2 µM of both 13104_A (FAM) and 13104_C (HEX) probes, 2 µL template DNA and 1.8 μL nuclease-free water (Cytiva, Amersham Place, United Kingdom). Optimal thermal cycling conditions were as follows: initial denaturation step at 95 °C for 30 s, followed by 40 cycles including a denaturation step at 95 °C for 5 s and 58 °C 30 s in the annealing step. On the other hand, for the duplex 13124-probe qPCR assay some variations were required: 0.3 µM of both 13124_A (Cy5) and 13124_G (FAM) probes, 3.6 µL template DNA and no nuclease-free water were employed. Moreover, 60 °C during 45 s were applied during the annealing step of the thermal cycler.

## Supplementary Information

Supplementary Information.

## Data Availability

All data generated or analysed during this study are included in this published article (and its Supplementary Information files).

## References

[1] Miranda G, Bianchi L, Krupova Z, Trossat P, Martin P (2020). An improved LC–MS method to profile molecular diversity and quantify the six main bovine milk proteins, including genetic and splicing variants as well as post-translationally modified isoforms. Food Chem. X.

[2] Caroli AM, Chessa S, Erhardt GJ (2009). Invited review: Milk protein polymorphisms in cattle: Effect on animal breeding and human nutrition. J. Dairy Sci..

[3] Azevedo A (2008). Genetic polymorphism of the kappa-casein gene in Brazilian cattle. Genet. Mol. Res..

[4] Kovalchuk S, Tagmazyan A, Klimov E (2019). A novel test system for genotyping rs43703016 single-nucleotide substitutions in the bovine CSN3 gene. Annu. Res. Rev. Biol..

[5] Vidović V (2013). Heritability and correlations of milk traits. Mljekarstvo.

[6] Deb R (2014). Genetic polymorphism and association of kappa-casein gene with milk production traits among Frieswal (HF × Sahiwal) cross breed of Indian origin. Iran. J. Vet. Res..

[7] Vafin RR, Gilmanov KK (2021). Real-time PCR technology for cattle genotyping by A and B kappa-casein gene alleles. Ser. Chem. Technol..

[8] Djedović R (2015). Relationship between genetic polymorphism of κ-casein and quantitative milk yield traits in cattle breeds and crossbreds in Serbia. Genetika.

[9] Boland M, Macgibbon A, Hill J (2001). Designer milks for the new millennium. Livest. Prod. Sci..

[10] Dalgleish DG, Corredig M (2012). The structure of the casein micelle of milk and its changes during processing. Annu. Rev. Food Sci. Technol..

[11] Fonseca PAS (2013). A new tetra-primer ARMS-PCR for genotyping bovine kappa-casein polymorphisms. Genet. Mol. Res..

[12] Adamov N (2020). Allele and genotype frequencies of the kappa-casein (CSN3) locus in Macedonian Holstein-Friesian Cattle. Maced. Vet. Rev..

[13] Chessa S (2020). The effect of selection on casein genetic polymorphisms and haplotypes in Italian Holstein cattle. Ital. J. Anim. Sci..

[14] Sanchez MP, Fritz S, Patry C, Delacroix-Buchet A, Boichard D (2020). Frequencies of milk protein variants and haplotypes estimated from genotypes of more than 1 million bulls and cows of 12 French cattle breeds. J. Dairy Sci..

[15] Volkandari S, Indriawati I, Margawati E (2017). Genetic polymorphism of kappa-casein gene in Friesian Hostein: A basic selection of dairy cattle superiority. J. Indones. Trop. Anim. Agric..

[16] Hallén E, Wedholm A, Andrén A, Lundén A (2008). Effect of β-casein, κ-casein and β-lactoglobulin genotypes on concentration of milk protein variants. J. Anim. Breed. Genet..

[17] Tyulkin SV (2018). Technological properties of milk of cows with different genotypes of kappa-casein and beta-lactoglobulin. Foods Raw Mater..

[18] Kübarsepp I, Henno M, Viinalass H, Sabre D (2005). Effect of κ-casein and β-lactoglobulin genotypes on the milk rennet coagulation properties. Agron. Res..

[19] Arslan M (2020). A new primer for PCR-RFLP analysis of A and B genetic variant of bovine kappa-casein. Harran Üniversitesi Veteriner Fakültesi Dergisi.

[20] Miglior F (2017). A 100-year review: Identification and genetic selection of economically important traits in dairy cattle. J. Dairy Sci..

[21] Alim MA (2014). Effect of polymorphisms in the CSN3 (κ-casein) gene on milk production traits in Chinese Holstein Cattle. Mol. Biol. Rep..

[22] Cole JB, Eaglen SAE, Maltecca C, Mulder HA, Pryce JE (2020). The future of phenomics in dairy cattle breeding. Anim. Front..

[23] Potočnik, K. Selection adapted to local conditions has the possibility to improve the economy of small dairy cattle populations. In *20th International Congress on Biotechnology in Animal Reproduction (ICBAR)* (2015).

[24] Barroso A, Dunner S, Cañ J (1998). Technical Note: Detection of bovine kappa-casein variants A, B, C, and E by means of polymerase chain reaction-single strand conformation polymorphism (PCR-SSCP) 1. J. Anim. Sci..

[25] Rincó G, Medrano JF (2003). Single nucleotide polymorphism genotyping of bovine milk protein genes using the tetra-primer ARMS-PCR. J. Anim. Breed. Genet..

[26] Sulimova GE, Azari MA, Rostamzadeh J, Mohammad Abadi MR, Lazebny OE (2007). κ-casein gene (CSN3) allelic polymorphism in Russian cattle breeds and its information value as a genetic marker. Russ. J. Genet..

[27] Ilie DE, Neamț RI, Popescu C, Săplăcan G (2017). Preliminary report on CSN3 and LGB genes polymorphism among two Romanian cattle breeds. Sci. Pap. Anim. Sci. Biotechnol..

[28] Ilie DE, Cean A, Gavriliuc O, Carstea CA, Grădinaru AC (2013). High-resolution melting assay as a tool for identification of CSN3 genotypes in cattle population. Sci. Pap. Anim. Sci. Biotechnol..

[29] Kyseľová J, Rychtářová J, Sztankóová Z, Czerneková V (2012). Simultaneous identification of CSN3 and LGB genotypes in cattle by high-resolution melting curve analysis. Livest. Sci..

[30] Puente-Lelievre C, Eischeid AC (2021). Development and validation of a duplex real-time PCR assay with locked nucleic acid (LNA) probes for the specific detection of allergenic walnut in complex food matrices. Food Control.

[31] You Y, Moreira BG, Behlke MA, Owczarzy R (2006). Design of LNA probes that improve mismatch discrimination. Nucleic Acids Res..

[32] Agbagwa IO, Datta S, Patil PG, Singh P, Nadarajan N (2012). A protocol for high-quality genomic DNA extraction from legumes. Genet. Mol. Res..

[33] Usman T, Yu Y, Liu C, Fan Z, Wang Y (2014). Comparison of methods for high quantity and quality genomic DNA extraction from raw cow milk. Genet. Mol. Res..

[34] Cremonesi P (2006). Technical note: Improved method for rapid DNA extraction of mastitis pathogens directly from milk. J. Dairy Sci..

[35] Liao J, Liu Y (2018). Purification procedures meaningfully influence DNA quantification in milk. LWT.

[36] Wassermann S (2020). A1/A2 β-Casein Charakterisierung mittels Real-Time-PCR.

[37] Desjardins P, Conklin D (2010). NanoDrop microvolume quantitation of nucleic acids. J. Vis. Exp..

[38] Sukumaran S (2010). Concentration determination of nucleic acids and proteins using the micro-volume Bio-Spec nano spectrophotometer. J. Vis. Exp..

[39] Conte J, Potoczniak MJ, Tobe SS (2018). Using synthetic oligonucleotides as standards in probe-based qPCR. Biotechniques.

[40] Taylor S, Wakem M, Dijkman G, Alsarraj M, Nguyen M (2010). A practical approach to RT-qPCR-Publishing data that conform to the MIQE guidelines. Methods.

[41] Baudy P (2019). A glance into the black box: Novel species-specific quantitative real-time PCR assays to disentangle aquatic hyphomycete community composition. Fungal Ecol..

[42] Zheng W (2019). Development and validation of quantitative real-time PCR for the detection of residual CHO host cell DNA and optimization of sample pretreatment method in biopharmaceutical products. Biol. Proced. Online.

[43] Forootan A (2017). Methods to determine limit of detection and limit of quantification in quantitative real-time PCR (qPCR). Biomol. Detect. Quantif..

[44] Chen X, Lu L, Xiong X, Xiong X, Liu Y (2020). Development of a real-time PCR assay for the identification and quantification of bovine ingredient in processed meat products. Sci. Rep..

[45] FDA. *Guidelines for the Validation of Analytical Methods for Nucleic Acid Sequence-Based Analysis of Food, Feed, Cosmetics and Veterinary Products*. www.fda.gov (2020).

[46] Giglioti R (2020). New high-sensitive rhAmp method for A1 allele detection in A2 milk samples. Food Chem..

[47] BOE (2013). Royal Decree 53/2013 of 1 February Establishing the Basic Rules Applicable to the Protection of Animals Used for Experimental and Other Scientific Purposes, Including Teaching.

[48] Ugozzoli LA, Latorra D, Pucket R, Arar K, Hamby K (2004). Real-time genotyping with oligonucleotide probes containing locked nucleic acids. Anal. Biochem..

[49] Champely, S. pwr: Basic Functions for Power Analysis. R package version 1.3-0, https://CRAN.R-project.org/package=pwr (2020).

[50] Pokorska J, Kułaj D, Dusza M, Żychlińska-Buczek J, Makulska J (2016). New rapid method of DNA isolation from milk somatic cells. Anim. Biotechnol..

[51] Jiménez-Montenegro, L., Mendizabal, J. A., Alfonso, L. & Urrutia, O. DNA extraction procedures and validation parameters of a real time PCR method to control milk containing only A2 β-casein. Food Control (2022). **(in revision).**

[52] Brugè F, Littarru GP, Silvestrini L, Mancuso T, Tiano L (2009). A novel real time PCR strategy to detect SOD3 SNP using LNA probes. Mutat. Res. Fundam. Mol. Mech. Mutagenesis.

[53] Latorra D, Campbell K, Wolter A, Hurley JM (2003). Enhanced allele-specific PCR discrimination in SNP genotyping using 3′ locked nucleic acid (LNA) primers. Hum. Mutat..

[54] Codex Alimentarius (2010). Guidelines on Performance Criteria and Validation of Methods for Detection, Identification and Quantification of Specific DNA Sequences and Specific Proteins in Foods.

[55] Burd EM (2010). Validation of laboratory-developed molecular assays for infectious diseases. Clin. Microbiol. Rev..

[56] Friedman CS, Wight N, Crosson LM, White SJ, Strenge RM (2014). Validation of a quantitative PCR assay for detection and quantification of “*Candidatus Xenohaliotis californiensis*”. Dis. Aquat. Org..

[57] Bustin SA (2009). The MIQE guidelines: Minimum information for publication of quantitative real-time PCR experiments. Clin. Chem..

