# Supplementary material for: Development of a duplex qPCR assay with locked nucleic acid probes for A, B and E kappa-casein variants detection
Source: Sci Rep. 2022 Sep 30;12:16387. doi: 10.1038/s41598-022-20586-w (PMC9525573; doi:10.1038/s41598-022-20586-w)
Supplement: Supplementary file 1 — Supplementary Information. [file 41598_2022_20586_MOESM1_ESM.pdf]

Development of a duplex qPCR assay with locked nucleic acid probes for A, B and E kappa-casein variants detection

L. Jiménez-Montenegro, J.A. Mendizabal, L. Alfonso, L. Azparren, O. Urrutia*

IS-FOOD, School of Agricultural Engineering and Biosciences, Public University of Navarre (UPNA), Campus de Arrosadia, 31006 Pamplona, Spain

*olaia.urrutia@unavarra.es

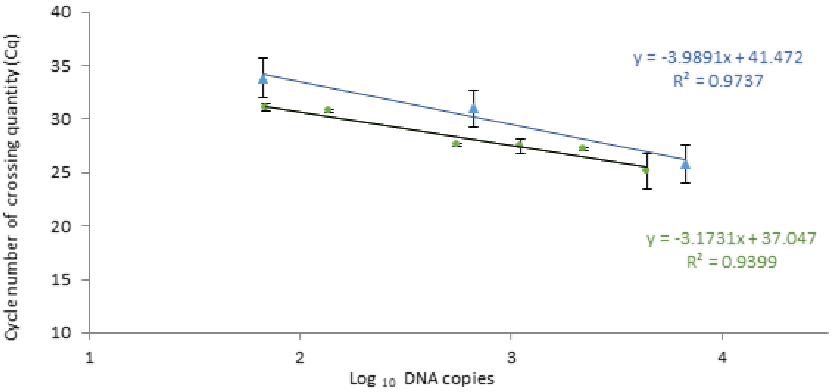

Figure S1. Duplex real-time PCR standard curves using the A synthetic reference DNA sample (blue colour) and the AA homozygous genotyped sample (green colour). The standard curves were made by plotting Cq values against log_{10} DNA copies/reaction of each type of sample. Each data point represents the averaged Cq value of three replicates.

Table S1.

Intra- and inter-assay repeatability of duplex real-time PCR assay for A, B and E allele discrimination of the *CSN3* gene using synthetic reference DNA samples of A, B and E alleles and 13124 and 13104 probes.

**13104 duplex qPCR assay[a]**									
					**Intra-assay repeatability[b]**		**Inter-assay repeatability[c]**		
Synthetic reference DNA	Run	Replicate	DNA copies/reaction[b,c]	Mean Cq	SD	CV (%)	Mean Cq	SD	CV (%)
A_gBlock	1	1	$6.70x10^6$	16.99	0.55	3.34			
		2	$6.70x10^6$	16.18					
		3	$6.70x10^6$	15.95					
	2	1	$6.70x10^6$	17.11	0.21	1.28	17.94	0.35	1.92
		2	$6.70x10^6$	16.71					
		3	$6.70x10^6$	16.76					
	3	1	$6.70x10^6$	21.43	0.95	4.60			
		2	$6.70x10^6$	20.79					
		3	$6.70x10^6$	19.57					

	1	1	6.70×10^{5}	19.34	0.55	2.92			
		2	6.70×10^{5}	18.36					
		3	6.70×10^{5}	19.30					
	2	1	6.70×10^{5}	20.12	0.02	0.09	20.85	0.79	3.79
		2	6.70×10^{5}	20.13					
		3	6.70×10^{5}	20.10					
	3	1	6.70×10^{5}	22.65	0.98	4.18			
		2	6.70×10^{5}	23.10					
		3	6.70×10^{5}	24.53					
	1	1	6.70×10^{4}	22.80	0.12	0.51			
		2	6.70×10^{4}	22.80					
		3	6.70×10^{4}	22.60					
	2	1	6.70×10^{4}	23.61	0.12	0.52	23.96	0.54	2.25
		2	6.70×10^{4}	23.52					
		3	6.70×10^{4}	23.36					
	3	1	6.70×10^{4}	25.78	0.14	0.55			
		2	6.70×10^{4}	25.69					
		3	6.70×10^{4}	25.50					
	1	1	6.70×10^{3}	24.11	0.26	1.08			
		2	6.70×10^{3}	24.29					
		3	6.70×10^{3}	24.63					
	2	1	6.70×10^{3}	25.05	0.38	1.50	25.83	0.69	2.65
		2	6.70×10^{3}	25.14					
		3	6.70×10^{3}	25.75					
	3	1	6.70×10^{3}	26.92	0.81	2.91			
		2	6.70×10^{3}	28.15					
		3	6.70×10^{3}	28.44					
	1	1	6.70×10^{2}	29.70	0.33	1.03			
		2	6.70×10^{2}	29.10					
		3	6.70×10^{2}	29.64					
	2	1	6.70×10^{2}	30.83	0.47	1.39	30.95	0.71	2.29
		2	6.70×10^{2}	30.67					
		3	6.70×10^{2}	29.95					
	3	1	6.70×10^{2}	32.86	0.82	2.31			
		2	6.70×10^{2}	33.74					
		3	6.70×10^{2}	32.09					
	1	1	6.70×10^{1}	32.14	0.05	0.16			
		2	6.70×10^{1}	32.15					
		3	6.70×10^{1}	32.05					
	2	1	6.70×10^{1}	33.20	0.35	1.05	33.81	1.05	3.11
		2	6.70×10^{1}	33.86					
		3	6.70×10^{1}	33.73					
	3	1	6.70×10^{1}	34.71	0.92	2.59			
		2	6.70×10^{1}	35.90					
		3	6.70×10^{1}	36.53					
	1	1	*&6.70×10^{0}	32.82	0.51	1.55			
		2	*&6.70×10^{0}	33.62					
		3	*&6.70×10^{0}	32.66					
	2	1	*&6.70×10^{0}	35.81	0.45	1.25	35.72	2.32	6.50
		2	*&6.70×10^{0}	36.68					
		3	*&6.70×10^{0}	36.46					
	3	1	*&6.70×10^{0}	37.25	1.03	2.73			
		2	*&6.70×10^{0}	39.01					
		3	*&6.70×10^{0}	37.20					
B_gBlock	1	1	6.70×10^{6}	15.88	0.19	1.19			
		2	6.70×10^{6}	15.80					
		3	6.70×10^{6}	15.52					
	2	1	6.70×10^{6}	16.18	0.05	0.34	15.99	0.27	1.71
		2	6.70×10^{6}	16.11					
		3	6.70×10^{6}	16.07					
	3	1	6.70×10^{6}	16.10	0.07	0.41			
		2	6.70×10^{6}	16.21					
		3	6.70×10^{6}	16.10					

	1	1	6.70x10^{5}	18.92	0.17	0.89			
		2	6.70x10^{5}	18.62					
		3	6.70x10^{5}	18.64					
	2	1	6.70x10^{5}	19.09	0.03	0.14	19.03	0.28	1.45
		2	6.70x10^{5}	19.12					
		3	6.70x10^{5}	19.14					
	3	1	6.70x10^{5}	19.27	0.03	0.17			
		2	6.70x10^{5}	19.20					
		3	6.70x10^{5}	19.24					
	1	1	6.70x10^{4}	22.54	0.04	0.18			
		2	6.70x10^{4}	22.48					
		3	6.70x10^{4}	22.56					
	2	1	6.70x10^{4}	22.74	0.03	0.12	22.69	0.14	0.60
		2	6.70x10^{4}	22.73					
		3	6.70x10^{4}	22.69					
	3	1	6.70x10^{4}	22.80	0.05	0.24			
		2	6.70x10^{4}	22.80					
		3	6.70x10^{4}	22.89					
	1	1	6.70x10^{3}	25.23	0.15	0.57			
		2	6.70x10^{3}	25.50					
		3	6.70x10^{3}	25.46					
	2	1	6.70x10^{3}	25.84	0.18	0.69	25.80	0.35	1.34
		2	6.70x10^{3}	25.73					
		3	6.70x10^{3}	26.08					
	3	1	6.70x10^{3}	25.99	0.13	0.48			
		2	6.70x10^{3}	26.20					
		3	6.70x10^{3}	26.20					
	1	1	6.70x10^{2}	29.26	0.09	0.32			
		2	6.70x10^{2}	29.39					
		3	6.70x10^{2}	29.20					
	2	1	6.70x10^{2}	29.39	0.23	0.78	29.49	0.18	0.59
		2	6.70x10^{2}	29.40					
		3	6.70x10^{2}	29.80					
	3	1	6.70x10^{2}	29.81	0.13	0.45			
		2	6.70x10^{2}	29.54					
		3	6.70x10^{2}	29.66					
	1	1	*6.70x10^{1}	32.95	0.40	1.24			
		2	*6.70x10^{1}	32.84					
		3	*6.70x10^{1}	32.20					
	2	1	*6.70x10^{1}	32.24	0.44	1.34	32.73	0.03	0.09
		2	*6.70x10^{1}	32.76					
		3	*6.70x10^{1}	33.11					
	3	1	*6.70x10^{1}	32.94	0.45	1.36			
		2	*6.70x10^{1}	32.33					
		3	*6.70x10^{1}	33.20					
	1	1	[&]6.70x10^{0}	ND	2.57	7.13			
		2	[&]6.70x10^{0}	37.83					
		3	[&]6.70x10^{0}	34.20					
	2	1	[&]6.70x10^{0}	37.11	1.03	2.87	36.23	0.04	0.11
		2	[&]6.70x10^{0}	36.05					
		3	[&]6.70x10^{0}	35.05					
	3	1	[&]6.70x10^{0}	36.78	1.35	3.67			
		2	[&]6.70x10^{0}	35.18					
		3	[&]6.70x10^{0}	37.85					
E_gBlock	1	1	6.70x10^{6}	15.76	0.83	5.47			
		2	6.70x10^{6}	15.56					
		3	6.70x10^{6}	14.23					
	2	1	6.70x10^{6}	16.52	0.29	1.78	16.99	0.96	5.65
		2	6.70x10^{6}	16.85					
		3	6.70x10^{6}	16.26					
	3	1	6.70x10^{6}	19.80	0.59	3.09			
		2	6.70x10^{6}	19.29					

		3	6.70x10^{6}	18.61					
	1	1	6.70x10^{5}	19.57	0.17	0.82			
		2	6.70x10^{5}	19.37					
		3	6.70x10^{5}	19.24					
	2	1	6.70x10^{5}	19.57	0.05	0.22	20.27	0.14	0.70
		2	6.70x10^{5}	19.64					
		3	6.70x10^{5}	19.56					
	3	1	6.70x10^{5}	22.16	0.31	1.36			
		2	6.70x10^{5}	21.55					
		3	6.70x10^{5}	21.78					
	1	1	6.70x10^{4}	22.46	0.23	1.01			
		2	6.70x10^{4}	22.72					
		3	6.70x10^{4}	22.26					
	2	1	6.70x10^{4}	23.18	0.13	0.58	23.58	0.42	1.78
		2	6.70x10^{4}	23.11					
		3	6.70x10^{4}	22.92					
	3	1	6.70x10^{4}	25.23	0.21	0.84			
		2	6.70x10^{4}	25.40					
		3	6.70x10^{4}	24.97					
	1	1	6.70x10^{3}	25.40	0.17	0.66			
		2	6.70x10^{3}	25.36					
		3	6.70x10^{3}	25.67					
	2	1	6.70x10^{3}	26.00	0.23	0.89	26.90	0.55	2.06
		2	6.70x10^{3}	26.46					
		3	6.70x10^{3}	26.32					
	3	1	6.70x10^{3}	29.16	0.21	0.71			
			6.70x10^{3}	28.76					
		2	6.70x10^{3}	29.00					
	1	1	6.70x10^{2}	30.06	0.46	1.56			
		2	6.70x10^{2}	29.54					
		3	6.70x10^{2}	29.13					
	2	1	6.70x10^{2}	30.39	0.10	0.33	30.98	0.49	1.58
		2	6.70x10^{2}	30.23					
		3	6.70x10^{2}	30.20					
	3	1	6.70x10^{2}	33.87	0.73	0.21			
		2	6.70x10^{2}	33.01					
		3	6.70x10^{2}	32.41					
	1	1	*6.70x10^{1}	32.10	0.23	0.70			
		2	*6.70x10^{1}	32.16					
		3	*6.70x10^{1}	31.74					
	2	1	*6.70x10^{1}	33.12	0.16	0.49	33.67	0.91	2.71
		2	*6.70x10^{1}	33.44					
		3	*6.70x10^{1}	33.32					
	3	1	*6.70x10^{1}	35.67	0.16	0.45			
		2	*6.70x10^{1}	35.89					
		3	*6.70x10^{1}	35.58					
	1	1	[&]6.70x10^{0}	33.48	0.15	0.44			
		2	[&]6.70x10^{0}	32.22					
		3	[&]6.70x10^{0}	33.46					
	2	1	[&]6.70x10^{0}	36.24	1.12	3.05	36.45	2.29	6.29
		2	[&]6.70x10^{0}	35.76					
		3	[&]6.70x10^{0}	37.89					
	3	1	[&]6.70x10^{0}	ND	0.63	1.61			
		2	[&]6.70x10^{0}	39.78					
		3	[&]6.70x10^{0}	38.88					
**13124 duplex qPCR assay[a]**									

				Intra-assay repeatability[b]			Inter-assay repeatability[c]		
Synthetic reference DNA	Run	Replicate	DNA copies/reaction[b,c]	Mean Cq	SD	CV (%)	Mean Cq	SD	CV (%)
A_gBlock	1	1	6.70x10^{6}	16.56	0.06	0.36			
		2	6.70x10^{6}	16.46					
		3	6.70x10^{6}	16.46					
	2	1	6.70x10^{6}	16.66	0.11	0.69	16.32	0.34	2.08
		2	6.70x10^{6}	16.48					
		3	6.70x10^{6}	16.45					
	3	1	6.70x10^{6}	15.80	0.12	0.74			
		2	6.70x10^{6}	16.03					
		3	6.70x10^{6}	15.94					
	1	1	6.70x10^{5}	19.94	0.01	0.07			
		2	6.70x10^{5}	19.97					
		3	6.70x10^{5}	19.96					
	2	1	6.70x10^{5}	19.85	0.04	0.22	19.69	0.35	1.79
		2	6.70x10^{5}	19.77					
		3	6.70x10^{5}	19.83					
	3	1	6.70x10^{5}	19.27	0.02	0.09			
		2	6.70x10^{5}	19.30					
		3	6.70x10^{5}	19.30					
	1	1	6.70x10^{4}	23.10	23.13	0.04			
		2	6.70x10^{4}	23.17					
		3	6.70x10^{4}	23.11					
	2	1	6.70x10^{4}	23.26	23.20	0.08	23.51	0.59	2.53
		2	6.70x10^{4}	23.24					
		3	6.70x10^{4}	23.11					
	3	1	6.70x10^{4}	22.70	24.19	2.62			
		2	6.70x10^{4}	22.66					
		3	6.70x10^{4}	27.22					
	1	1	6.70x10^{3}	24.47	0.02	0.09			
		2	6.70x10^{3}	24.50					
		3	6.70x10^{3}	24.46					
	2	1	6.70x10^{3}	24.48	0.04	0.18	24.37	0.23	0.94
		2	6.70x10^{3}	24.54					
		3	6.70x10^{3}	24.57					
	3	1	6.70x10^{3}	24.10	0.02	0.07			
		2	6.70x10^{3}	24.13					
		3	6.70x10^{3}	24.10					
	1	1	6.70x10^{2}	30.12	0.07	0.23			
		2	6.70x10^{2}	30.19					
		3	6.70x10^{2}	30.05					
	2	1	6.70x10^{2}	30.12	0.09	0.29	29.83	0.43	1.44
		2	6.70x10^{2}	29.99					
		3	6.70x10^{2}	29.95					
	3	1	6.70x10^{2}	29.40	0.06	0.21			
		2	6.70x10^{2}	29.29					
		3	6.70x10^{2}	29.31					
	1	1	6.70x10^{1}	29.67	2.12	6.60			
		2	6.70x10^{1}	33.47					
		3	6.70x10^{1}	33.19					
	2	1	6.70x10^{1}	33.09	0.13	0.40	32.70	0.57	1.74
		2	6.70x10^{1}	33.32					
		3	6.70x10^{1}	33.34					
	3	1	6.70x10^{1}	32.76	0.21	0.53			
		2	6.70x10^{1}	32.92					
		3	6.70x10^{1}	32.51					

	1	1	*&$6.70x10^{0}$	34.03	0.16	0.47			
		2	*&$6.70x10^{0}$	34.35					
		3	*&$6.70x10^{0}$	34.16					
	2	1	*&$6.70x10^{0}$	34.09	0.06	0.18	34.08	0.12	0.36
		2	*&$6.70x10^{0}$	34.17					
		3	*&$6.70x10^{0}$	34.05					
	3	1	*&$6.70x10^{0}$	34.11	0.64	0.01			
		2	*&$6.70x10^{0}$	33.70					
		3	*&$6.70x10^{0}$	34.03					
B_gBlock	1	1	$6.70x10^{6}$	17.15	0.06	0.38			
		2	$6.70x10^{6}$	17.17					
		3	$6.70x10^{6}$	17.27					
	2	1	$6.70x10^{6}$	17.34	0.08	0.47	17.02	0.35	2.06
		2	$6.70x10^{6}$	17.25					
		3	$6.70x10^{6}$	17.18					
	3	1	$6.70x10^{6}$	16.66	0.04	0.22			
		2	$6.70x10^{6}$	16.59					
		3	$6.70x10^{6}$	16.61					
	1	1	$6.70x10^{5}$	20.38	0.05	0.24			
		2	$6.70x10^{5}$	20.45					
		3	$6.70x10^{5}$	20.36					
	2	1	$6.70x10^{5}$	20.38	0.06	0.28	20.26	0.20	1.01
		2	$6.70x10^{5}$	20.40					
		3	$6.70x10^{5}$	20.29					
	3	1	$6.70x10^{5}$	20.04	0.02	0.09			
		2	$6.70x10^{5}$	20.01					
		3	$6.70x10^{5}$	20.03					
	1	1	$6.70x10^{4}$	23.88	0.08	0.35			
		2	$6.70x10^{4}$	23.95					
		3	$6.70x10^{4}$	24.04					
	2	1	$6.70x10^{4}$	23.93	0.07	0.29	23.78	0.34	1.44
		2	$6.70x10^{4}$	24.00					
		3	$6.70x10^{4}$	24.07					
	3	1	$6.70x10^{4}$	23.39	0.04	0.16			
		2	$6.70x10^{4}$	23.34					
		3	$6.70x10^{4}$	23.42					
	1	1	$6.70x10^{3}$	26.75	0.18	0.68			
		2	$6.70x10^{3}$	27.09					
		3	$6.70x10^{3}$	27.05					
	2	1	$6.70x10^{3}$	27.01	0.02	0.06	26.83	0.29	1.09
		2	$6.70x10^{3}$	27.04					
		3	$6.70x10^{3}$	27.03					
	3	1	$6.70x10^{3}$	26.41	0.07	0.26			
		2	$6.70x10^{3}$	26.51					
		3	$6.70x10^{3}$	26.55					
	1	1	$6.70x10^{2}$	30.70	0.08				
		2	$6.70x10^{2}$	30.60					
		3	$6.70x10^{2}$	30.54					
	2	1	$6.70x10^{2}$	30.76	0.08	0.27	30.49	0.27	0.90
		2	$6.70x10^{2}$	30.59					
		3	$6.70x10^{2}$	30.67					
	3	1	$6.70x10^{2}$	30.28	0.11				
		2	$6.70x10^{2}$	30.17					
		3	$6.70x10^{2}$	30.06					
	1	1	$6.70x10^{1}$	33.85	0.22	0.64			
		2	$6.70x10^{1}$	33.92					
		3	$6.70x10^{1}$	34.26					
	2	1	$6.70x10^{1}$	33.81	0.14	0.43	33.61	0.42	1.25

		2	6.70x10^{1}	33.52					
		3	6.70x10^{1}	33.63					
	3	1	6.70x10^{1}	33.46	0.24	0.74			
		2	6.70x10^{1}	33.04					
		3	6.70x10^{1}	33.03					
	1	1	*&6.70x10^{0}	34.33	0.22	0.65			
		2	*&6.70x10^{0}	34.10					
		3	*&6.70x10^{0}	33.89					
	2	1	*&6.70x10^{0}	34.33	0.09	0.26	34.24	0.13	0.38
		2	*&6.70x10^{0}	34.15					
		3	*&6.70x10^{0}	34.26					
	3	1	*&6.70x10^{0}	34.07	0.48	1.41			
		2	*&6.70x10^{0}	34.92					
		3	*&6.70x10^{0}	34.10					
E_gBlock	1	1	6.70x10^{6}	17.11	0.19	1.09			
		2	6.70x10^{6}	17.15					
		3	6.70x10^{6}	16.81					
	2	1	6.70x10^{6}	17.73	0.33	1.91	17.30	0.24	1.41
		2	6.70x10^{6}	17.47					
		3	6.70x10^{6}	17.07					
	3	1	6.70x10^{6}	17.85	0.33	1.90			
		2	6.70x10^{6}	17.31					
		3	6.70x10^{6}	17.24					
	1	1	6.70x10^{5}	20.00	0.20	1.00			
		2	6.70x10^{5}	20.03					
		3	6.70x10^{5}	20.36					
	2	1	6.70x10^{5}	20.31	0.11	0.53	20.36	0.24	1.19
		2	6.70x10^{5}	20.47					
		3	6.70x10^{5}	20.26					
	3	1	6.70x10^{5}	20.62	0.02	0.09			
		2	6.70x10^{5}	20.60					
		3	6.70x10^{5}	20.63					
	1	1	6.70x10^{4}	23.33	0.28	1.20			
		2	6.70x10^{4}	23.78					
		3	6.70x10^{4}	23.26					
	2	1	6.70x10^{4}	23.47	0.15	0.63	23.59	0.11	0.47
		2	6.70x10^{4}	23.65					
		3	6.70x10^{4}	23.77					
	3	1	6.70x10^{4}	23.88	0.20	0.84			
		2	6.70x10^{4}	23.49					
		3	6.70x10^{4}	23.63					
	1	1	6.70x10^{3}	26.96	0.65	2.40			
		2	6.70x10^{3}	26.73					
		3	6.70x10^{3}	27.96					
	2	1	6.70x10^{3}	27.31	0.00	0.01	27.26	0.05	0.17
		2	6.70x10^{3}	27.31					
		3	6.70x10^{3}	27.31					
	3	1	6.70x10^{3}	27.24	0.05	0.17			
		2	6.70x10^{3}	27.20					
		3	6.70x10^{3}	27.29					
	1	1	6.70x10^{2}	31.28	5.41	19.40			
		2	6.70x10^{2}	21.63					
		3	6.70x10^{2}	30.68					
	2	1	6.70x10^{2}	32.03	0.61	1.95	30.16	1.99	6.59
		2	6.70x10^{2}	31.22					
		3	6.70x10^{2}	30.84					
	3	1	6.70x10^{2}	31.84	0.60	1.91			
		2	6.70x10^{2}	31.26					

	3	6.70x10^{2}	30.65					
1	1	6.70x10^{1}	35.00	0.63	1.83			
	2	6.70x10^{1}	34.15					
	3	6.70x10^{1}	33.77					
2	1	6.70x10^{1}	33.54	0.11	0.32	33.94	0.34	1.01
	2	6.70x10^{1}	33.75					
	3	6.70x10^{1}	33.61					
3	1	6.70x10^{1}	34.29	0.36	1.06			
	2	6.70x10^{1}	33.68					
	3	6.70x10^{1}	33.66					
1	1	*&6.70x10^{0}	36.73	1.59	4.31			
	2	*&6.70x10^{0}	35.38					
	3	*&6.70x10^{0}	38.55					
2	1	*&6.70x10^{0}	37.45	0.11	0.29	37.45	0.51	1.37
	2	*&6.70x10^{0}	37.58					
	3	*&6.70x10^{0}	37.67					
3	1	*&6.70x10^{0}	38.01	0.22	0.58			
	2	*&6.70x10^{0}	37.64					
	3	*&6.70x10^{0}	38.03					

Cq = Cycle number of crossing quantity; CV = coefficient of variation; ND = not determined; SD = standard deviation.

[a] The 13104 probes were labelled with FAM reporter (A and E allele detection) and HEX reporter (B allele detection), respectively. The 13124 probes were labelled with Cy5 reporter (A and B allele detection) and FAM reporter (E allele detection), respectively.

[b] Three replicates were done in the same run for each standard dilution.

[c] Three runs were done for each standard dilution (9 replicates in total for each standard dilution).

[&] LOQ value: lowest DNA copies of the linear dynamic range.

* LOD value of each duplex qPCR assay for each synthetic gBlock.

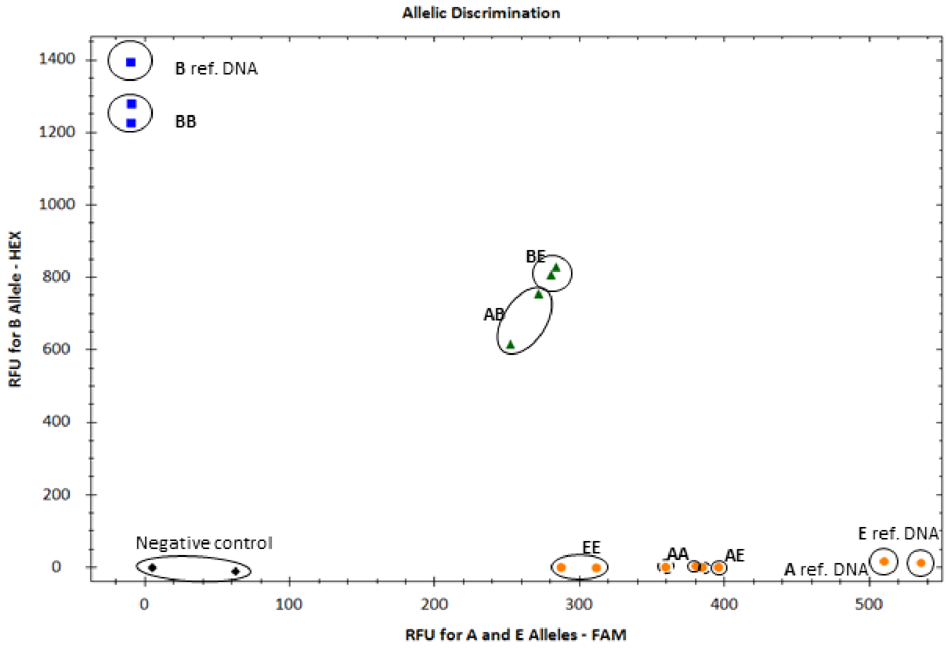

Figure S2. Applicability assays. Allelic discrimination graphs of 13104-probe PCR assay for AA, AB, BB, BE and EE genotyped DNA samples from milk of genotyped animals. Nonspecific amplifications were not observed. The blue dots represent BB genotype (HEX reporter), the greens dots represent heterozygous AB or BE genotypes, the yellow dots represent the heterozygous AE and the homozygous AA and EE genotypes (FAM reporter); the black dots represent negative control (NTC). A, B and E synthetic reference samples are used as a positive control

of the assay. Data were plotted using RFU of the FAM reporter dye of A and E alleles on the x axis and using RFU of the HEX reporter dye of B allele on the y axis. RFU = Relative Fluorescence Units.

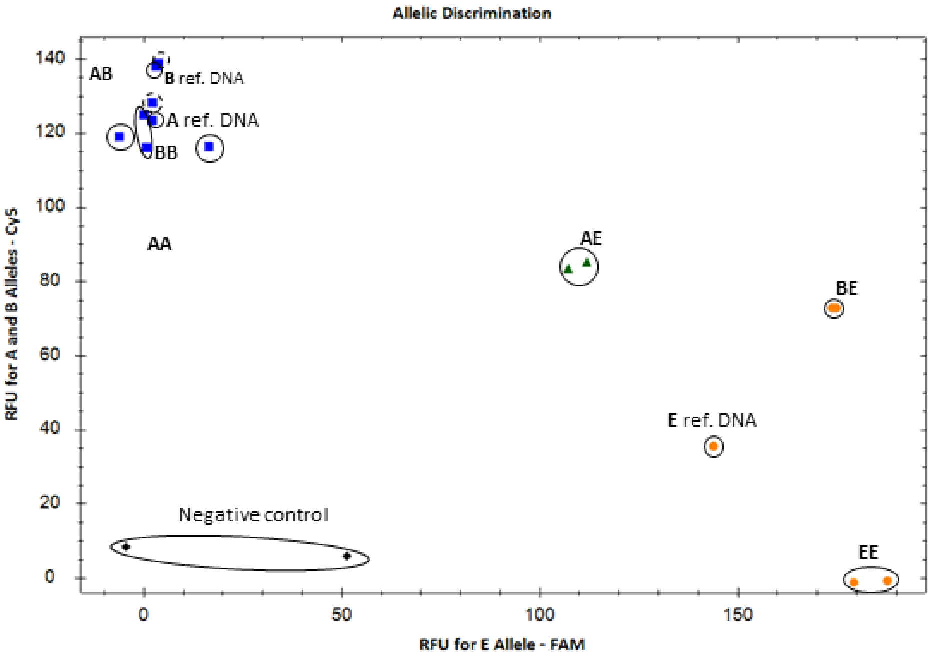

Figure S3. Allelic discrimination graph of 13124-probe PCR assay with AA, AB, BB, BE and EE genotyped DNA samples from milk genotyped animals. Blue dots represent the heterozygous AB genotype and the homozygous AA and BB genotypes (Cy5 reporter), the greens dots represent heterozygous AE or BE genotypes, yellow dots represent the homozygous EE genotype; black dots represent negative control (NTC). A, B and E synthetic reference samples are used as a positive control of the assay. Data were plotted on the x axis using RFU of the FAM reporter dye of E allele, and on the y axis using RFU of the Cy5 reporter dye of A and B allele. RFU = Relative Fluorescence Units.

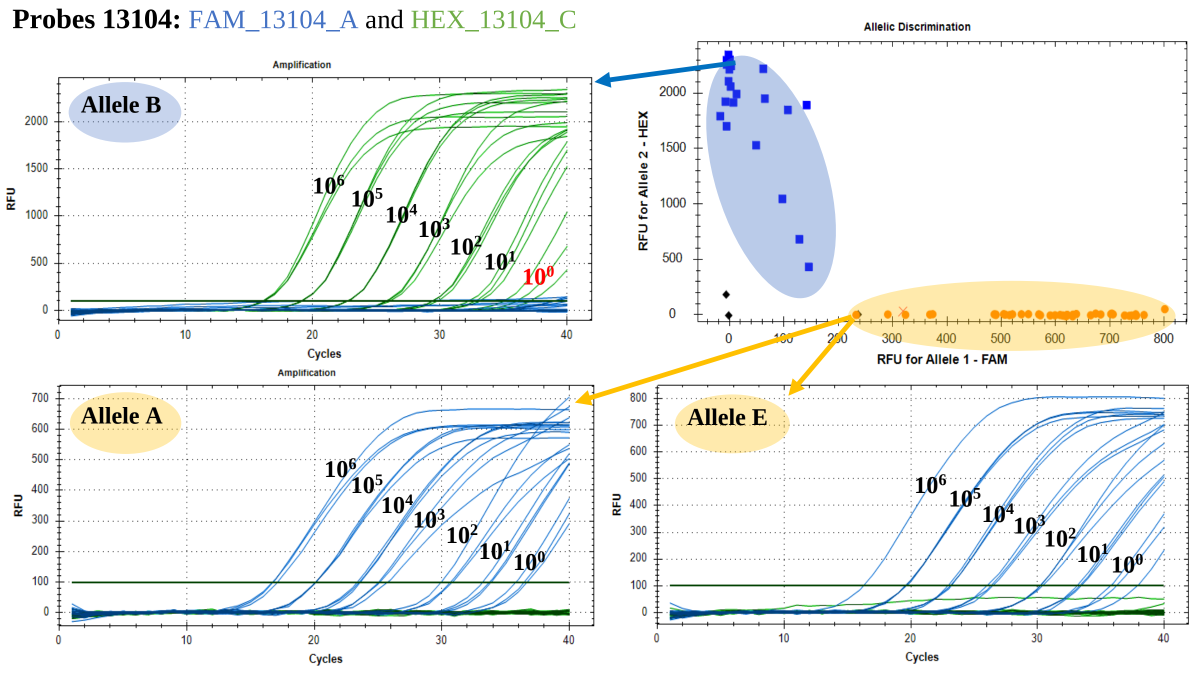

Figure S4. Amplification curves (RFU *vs.* cycle number) and allelic discrimination plot (RFU of the HEX fluorophore *vs.* RFU of the FAM fluorophore) for 13104 probes. The FAM_13104_A probe emits the blue signal and is specific for the A and E alleles, while the HEX_13104_C probe emits the green signal and is specific for the B

variant. Discarded dilutions are marked in red. This pair makes it possible to differentiate alleles A and E ("●"), from allele B ("■"). The "♦" values correspond to negative control.

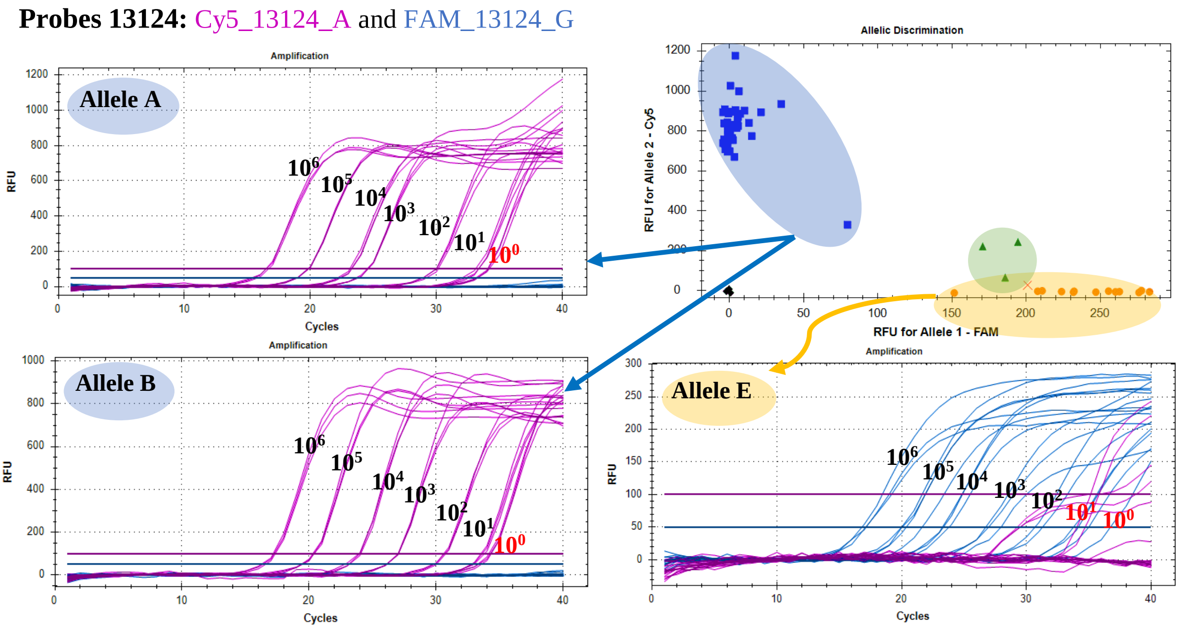

Figure S5. Amplification curves (RFU *vs.* cycle number) and allelic discrimination plot (RFU of the HEX fluorophore *vs.* RFU of the FAM fluorophore) for 13124 probes. The Cy5_13124_A probe emits the purple signal and is specific for the A and B alleles, while the FAM_13124_G probe emits the blue signal and is specific for the E allele. Discarded dilutions are marked in red. This pair makes it possible to differentiate allele E ("●"), from the other two alleles A and B ("■"). The "♦" values correspond to negative control.

S1. DNA isolation from milk somatic cells and hair samples

Milk samples pre-treatment and pre-lysis step

Fresh milk samples were pre-treated based on the method described by Yap et al. (2020). Briefly, 2x10 ml milk samples were centrifuged at 4500 g for 20 min at 4°C, the fat was removed by a sterile spatula and the supernatant was discarded. The two somatic cell pellets were pooled and resuspended in 800 µl phosphate-buffered saline (PBS, pH 7.4), before centrifugation at 4500 g for 1 min at room temperature. Subsequently, the supernatant was discarded and the pellet was washed twice in 1 ml of PBS. The somatic cells pellet was then stored at 20°C for 20 min. Pre-lysis step of unfreeze pellets was carried out according to *NucleoSpin Tissue* kit instructions. Unfreeze pellets were resuspended in 180 µL of T1 buffer, and then, 25 µl of Proteinase K were added, vortexed and incubated at 56°C for 2 hours. After pre-lysis, the DNA extraction continued with the lysis step described below.

Hair samples pre-lysis step

Hair roots from each individual sample was put into a 1.5 ml microcentrifuge tube. After that, two pre-lysis steps were carried out according to the *NucleoSpin Tissue* manufacturer's instructions. Firstly, 360 µL of Buffer T1 were added, then, a rapid freezing step with nitrogen liquid followed by a subsequent thawing in a thermoblock at 56 ºC were performed four different times. In the second pre-lysis step, 25 µl of Proteinase K were added, vortex mixing was performed and the samples were incubated at 56°C overnight. After pre-lysis, the DNA extraction continued with the lysis step described below.

NucleoSpin Tissue kit

DNA extraction from milk somatic cells and from hair samples was performed using the commercial *NucleoSpin Tissue* kit (Machenerey-Nagel) (Psifidi et al., 2010), following the manufacturer's instructions. After incubation, for lysis step, 200 µL of lysis Buffer B3 were added to the samples and they were incubated at 70°C for 10 min. Subsequently, to adjust DNA binding conditions, 210 µl of cold ethanol (96-100%) was added. Samples were then added to the *NucleoSpin Tissue* columns kit and centrifuged 1 min at 11000 g. The eluate was discarded. The aim of this last step was to remove the remaining components that were not DNA. Furthermore, this DNA purification was repeat, but this time, by adding 500 µl of Buffer BW. A second wash of the silica membrane was performed with 600 µL of Buffer B5 with ethanol and centrifuged again at 11000 g for 1 min. This last wash was performed in duplicate. To dry the silica membrane and remove residual ethanol from the silica membrane, the column was centrifuged at 11000 g for 1 minute. Finally, once all residues were removed, the DNA was eluted from the silica membrane into a 1.5 mL microcentrifuge tube. For this, 50 µL of Elution Buffer BE (preheated to 70°C) was used, incubated at room temperature for 1 minute and centrifuged for 1 minute at 11000 g. Each 1.5 ml microcentrifuge tube should contain approximately 50 µL of extracted DNA.

**Target**	**Oligonucleotide**	**Sequence (5'-3')[1]**	**Amplicon size (bp)**
CSN2	Forward primer	CAGTCTCTAGTCTATCCCTTCC	74
	Reverse primer	GTTTGAGTAAGAGGAGGGATGT	
CSN2	A2 allele: CD.GT.QWDC2715.6.1 WT	AAGTCAGCCTGCTCAGGATAAAATCCAC CCCTTTGCCCAGACACAGTCTCTAGTCTA TCCCTTCCCTGGGCCCATCCCTAACAGCC TCCCACAAAACATCCCTCCTCTTACTCAA ACCCCTGTGGTGGTGCCG	

Table S2. Primers and synthetic DNA gBlock Gene Fragment used for DNA amplificability evaluation.

**Target**	**gBlocks® Gene Fragments**	**Sequence (5'-3')[1]**
CSN3	A_gBlock	ATTGCTAGTGGTGAGCCTACAAGTACACCTACCACCGAAGCAGTAGAGAG CACTGTAGCTACTCTAGAAGATTCTCCAGAAGTTATTGAGAGCCCACCTGA GATCAACACAGTCCAAGTTACTTCAACTGCAGTCTAAAAACTCT
	B_gBlock	ATTGCTAGTGGTGAGCCTACAAGTACACCTACCACCGAAGCAGTAGAGAG CACTGTAGCTACTCTAGAAGCTTCTCCAGAAGTTATTGAGAGCCCACCTGA GATCAACACAGTCCAAGTTACTTCAACTGCAGTCTAAAAACTCT
	E_gBlock	ATTGCTAGTGGTGAGCCTACAAGTACACCTACCACCGAAGCAGTAGAGAG CACTGTAGCTACTCTAGAAGATTCTCCAGAAGTTATTGAGGGCCCACCTGA GATCAACACAGTCCAAGTTACTTCAACTGCAGTCTAAAAACTCT

Table S3. A, B and E synthetic DNA gBlocks Gene Fragment of the *CSN3* gene. [1]A, B and E K-casein polymorphisms are represented in red.

S2. DNA copy number calculation

Standard curves were created by plotting the Cq against the $\log_{10}$ of the DNA copy number[59]. The copy number for each synthetic reference DNA sample (A, B and E) were calculated according to the manufacturer instructions (IDT, Coralville, USA) with the following formula:

$$Number\ of\ copies\ for\ reference\ DNA\ samples = C\left(\frac{ng}{uL}\right) x\ M\left(\frac{fmol}{ng}\right) x\left(\frac{1x10^{-15}\,mol}{fmol}\right) x\ 6.022x10^{23}$$

where C is the concentration in ng/µL of the synthetic reference DNA samples (10 ng/µL), M is the molecular weight in fmol/ng (11.18 fmol/ng), and 6.022×10^{23} is the Avogadro constant.

In the case of AA genotyped DNA copy number were calculated as described previously[40,59]:

$$Number\ of\ copies\ for\ genotyped\ DNA\ samples = \frac{Amount\ of\ DNA\ (ng)\ x\ 6.022x10^{23}}{genome\ size\ (pb)\ x\ 10^{9}\frac{ng}{g}\ x\ mass\ of\ bp}$$

where 6.022×10^{23} is the Avogadro constant, bovine genome size was considered to be 3 billion base pairs and the average molecular mass per base pair was defined as 660 g/mol.
